# SARS-Cov-2 incubation period according to vaccination status during the fifth COVID-19 wave in a tertiary-care center in Spain: a cohort study

**DOI:** 10.1186/s12879-022-07822-4

**Published:** 2022-11-09

**Authors:** Jordi Cortés Martínez, Daewoo Pak, Gabriela Abelenda-Alonso, Klaus Langohr, Jing Ning, Alexander Rombauts, Mireia Colom, Yu Shen, Guadalupe Gómez Melis

**Affiliations:** 1 Department of Statistics and Operations Research, Universitat Politècnica de Catalunya, BarcelonaTech (UPC), 08034 Barcelona, Spain; 2 Division of Data Science, Yonsei University, Wonju, 26493 Korea; 3 Department of Infectious Diseases, Bellvitge University Hospital, 08907 L’Hospitalet De Llobregat, Spain; 4 Bellvitge Biomedical Research Institute (IDIBELL), 08907 L’Hospitalet De Llobregat, Spain; 5 Department of Biostatistics, The University of Texas, MD Anderson Cancer Center, Houston, TX 77030 USA

**Keywords:** COVID-19, Delta variant, Generalized odds-rate class of models, Incubation period, Infectious disease, Adenovirus-based vaccine

## Abstract

**Background:**

The incubation period of an infectious disease is defined as the elapsed time between the exposure to the pathogen and the onset of symptoms. Although both the mRNA-based and the adenoviral vector-based vaccines have shown to be effective, there have been raising concerns regarding possible decreases in vaccine effectiveness for new variants and variations in the incubation period.

**Methods:**

We conducted a unicentric observational study at the Hospital Universitari de Bellvitge, Barcelona, using a structured telephone survey performed by trained interviewers to estimate the incubation period of the SARS-CoV-2 Delta variant in a cohort of Spanish hospitalized patients. The distribution of the incubation period was estimated using the generalized odds-rate class of regression models.

**Results:**

From 406 surveyed patients, 242 provided adequate information to be included in the analysis. The median incubation period was 2.8 days (95%CI: 2.5–3.1) and no differences between vaccinated and unvaccinated patients were found. Sex and age are neither shown not to be significantly related to the COVID-19 incubation time.

**Conclusions:**

Knowing the incubation period is crucial for controlling the spread of an infectious disease: decisions on the duration of the quarantine or on the periods of active monitoring of people who have been at high risk of exposure depend on the length of the incubation period. Furthermore, its probability distribution is a key element for predicting the prevalence and the incidence of the disease.

**Supplementary Information:**

The online version contains supplementary material available at 10.1186/s12879-022-07822-4.

## Background

New Severe Acute Respiratory Syndrome Coronavirus 2 (SARS-CoV-2) variants with higher transmissibility, pathogenicity, and partial resistance to neutralization by the serum antibodies of vaccinated and convalescent individuals have emerged [1–3]. Although both the mRNA-based (mainly, Pfizer and Moderna) and the adenoviral vector-based (mainly, Johnson & Johnson and AstraZeneca) vaccines have shown to be effective, there has been raising concerns regarding possible decreases in vaccine effectiveness for new variants and variations in the incubation period.

The incubation period of an infectious disease is defined as the period from exposure to symptom onset. Knowing the incubation period is crucial for controlling the spread of an infectious disease: decisions on the duration of the quarantine or on the periods of active monitoring of people who have been at high risk of exposure depend on the length of the incubation period. Furthermore, the probability distribution of the incubation period is a key element for predicting the prevalence and the incidence of the disease.

To our knowledge, there is little evidence on the factors that may influence the incubation time of Sars-CoV2. Some studies [4, 5] showed that older people tended to present symptoms later after exposure to the virus in early variants. Daley et al. [6] conducted a systematic review to collect information about the potential influence of age, biological sex, and location on the incubation period at the pandemic’s outbreak. In their study the only relevant difference was found on the incubation times of different Chinese regions. Marks et al. [7] showed that there was an inverse correlation between viral load and incubation time. Related to this last point, Kaslow et al. [8] commented that vaccinated individuals may present longer times due to greater immune protection but without providing evidence for the specific case of COVID-19. Other studies, such as the one by Águila-Mejía et al. [9] found no significant differences in incubation times comparing vaccinated versus unvaccinated people, but they did not distinguish between different types of vaccine.

Despite having previously analyzed the incubation period of the original Wuhan strain [10] and several variants, mainly Alpha or Delta [11] and the influential factors, no studies have yet been specifically designed to estimate the incubation period depending on the vaccination status and the type of vaccine used. The aim of this study was to determine if the incubation period of the SARS-CoV-2 Delta variant differs between the vaccinated and unvaccinated patients and to analyze the incubation period depending on the type of vaccine administered.

## Methods

### Data collection

We conducted an observational unicentric study at the Hospital Universitari de Bellvitge, Barcelona, using a structured telephone survey performed by trained interviewers to estimate the COVID-19 incubation period. Although no test was done to determine the Sars-CoV-2 variant of admitted patients, the Delta variant is assumed due to the high prevalence of this variant during the period of data hospitalization. Between July 6th and December 13th 2021, we assessed all subjects older than 18 years admitted to the hospital with PCR-proven SARS-CoV-2 first episode infection. All included patients gave informed consent to participate in the study.

The required number of patients surveyed to achieve a precision of ± 2 days in the 95% confidence interval (CI) for the 75% percentile of the incubation time and assuming 20% of non-responders was 378. The determination of the sample size requires an estimation of the variability of the incubation time estimates based on interval-censored data. We have used the estimated variability in the percentiles provided by Pak et al. [4] (see Additional file 1: Appendix 1 for more details).

The telephone survey consisted of pre-designed questions to determine the moment of exposure in order to minimize variability. A case report form (CRF) was specifically designed for the study and it was implemented in the Redcap tool [12]. The main variables collected were the period of exposure to the infection and symptom onset dates. Other variables included were age, gender, vaccination status and type of vaccine administered (see Additional file 1: Appendix 2).

### Statistical analysis

The distribution of the COVID-19 incubation period was estimated with the generalized odds-rate class of regression models, which includes the log-logistic proportional odds model and the Weibull proportional hazards model as special cases [13]. The probability of an incubation time larger than a time *t*, denoted by $S\left(t|x\right)$, is given by

$$S\left(t|x\right)={\left\{1+\rho {\left(\frac{t}{\lambda }\right)}^{\phi }{e}^{{x}^{T}\beta }\right\}}^{-\frac{1}{\rho }}$$

where $\rho , \lambda ,$ $\phi$, and $\beta$ are model parameters and *x* is the covariate vector including patient’s age, sex and vaccination-related variables. The estimated median incubation time is obtained by solving $S\left(t|x\right)=0.5$ given the estimated coefficients and a specific set of covariates.

All the surveyed patients reported the exact date (${D}_{sym}$) of symptoms onset, but many of them neither remembered the exact date on which they were exposed nor were they even able to limit the dates of said exposure. These patients did not provide relevant information on incubation time and therefore, they were excluded from the analysis. Among the remaining patients we distinguished three levels of information depending on the censoring pattern:

- *Pattern I.* The patient knew the exact date (${D}_{exp}$) that he/she was exposed to the SARS-CoV-2. In this case the incubation time, *T*, is exactly observed and equal to (${D}_{sym}-{D}_{exp}$).
- *Pattern II.* The patient knew that he/she was exposed to the virus between day ${D}_{expL}$ and day${D}_{expU}$. In this case, the incubation time, T, is between ${D}_{sym}-{D}_{expU} \; and \; { D}_{sym}-{D}_{expL}$, this is what is known as an interval censored data.
- *Pattern III.* The patient only knew that he/she was exposed before day ${D}_{expU}$. The incubation time is, hence, larger than ${D}_{sym}-{D}_{expU}$, leading to a right-censored data.

The maximum number of observed incubation days among the patients from Pattern I reporting exact data was equal to 13 days. Based on this figure, we have set a maximum incubation period of 14 days. This would imply that ${D}_{sym}-{D}_{expL}\le 14$ and that the incubation times among the patients from Pattern III are restricted to the interval$\left[{D}_{sym}-{D}_{expU} , 14\right]$.

We tested all model combinations with the collected covariates and chose the best one according to the Akaike Information Criteria (AIC). We performed two sensitivity analyses regarding the models: (1) only including the patients with the censoring pattern I (known exact date of exposure); and (2) using a longer maximum incubation period of 21 days (instead of 14 days). All statistical analyses were performed with the statistical software R, version 4.1.2. (Vienna, Austria; https://www.r-project.org).

## Results

**Fig. 1:**
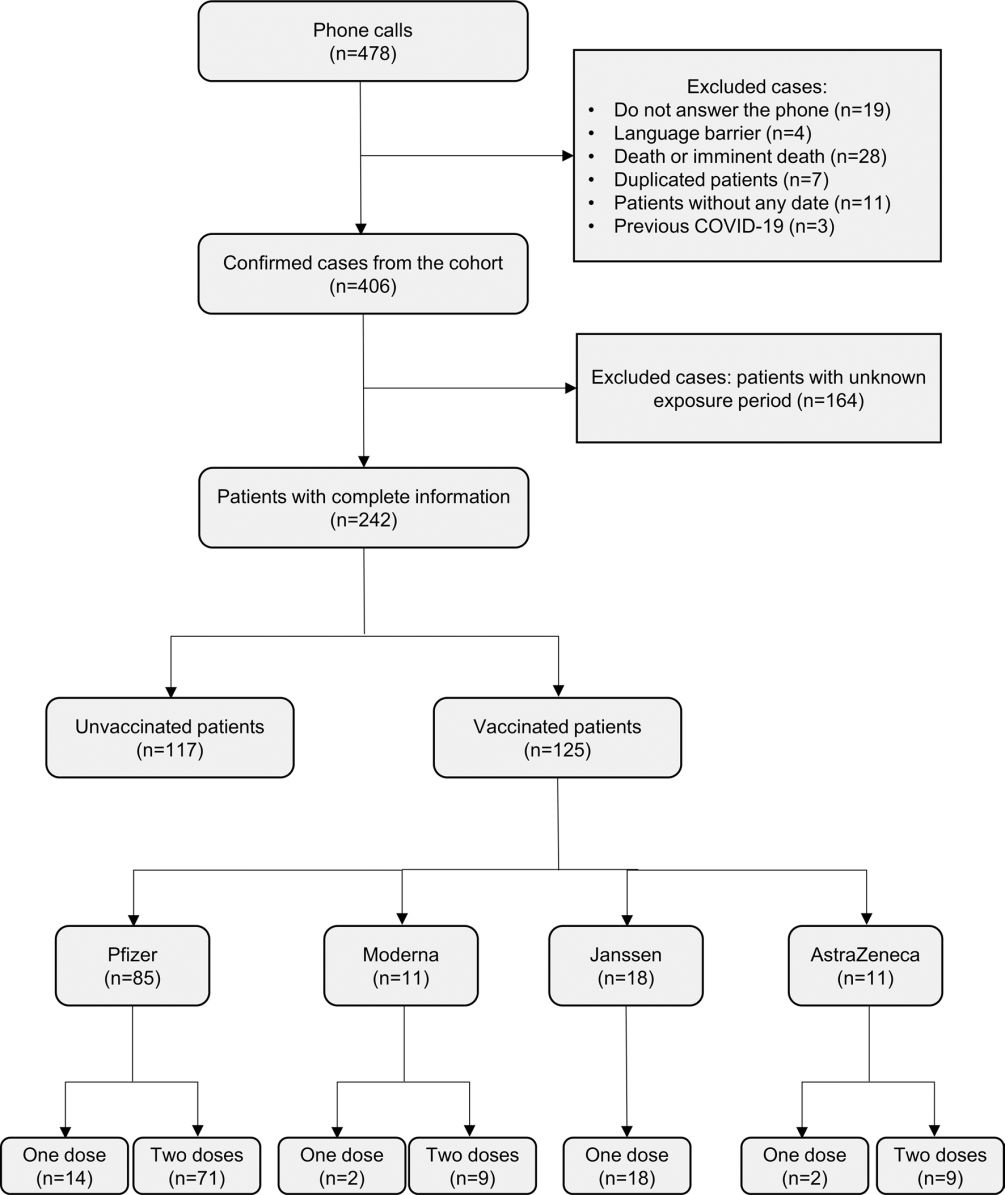
Flow diagram of confirmed cases for data analysis

A total of 478 patients were called by phone from July 6th to December 13th 2021, but only 406 of them were eligible for the study (see Fig. 1). Regarding SARS-CoV-2 exposure, 64 (16%) patients provided an exact date of contact with other COVID-19 symptomatic people (Pattern I) and 178 (44%) patients provided a complete or partial interval of exposure dates (Patterns II and III). The remaining 164 (40%) did not report any information regarding the potential exposure to the virus.

Among the 242 patients included in the analysis, the mean age was 58.0 years (± 19.9), 59.5% were male, and 51.7% received at least one dose of vaccine. From these, 29 (23%) received adenovirus-based vaccines (n = 18 for Janssen; n = 11 for Astrazeneca) while 96 (77%) were vaccinated with mRNA vaccines (n = 85 for Pfizer; n = 11 for Moderna). Table 1 shows the baseline information of included and excluded patients.

**Table 1:** Summary statistics of patients excluded/included in the analysis

	Excluded	Included
	*N* = 164	*N* = 242
Age	61.1 (17.6)	58.0 (19.9)
Age (grouped)		
18–29 years	5 (3.05%)	15 (6.20%)
30–39 years	21 (12.8%)	44 (18.2%)
40–49 years	21 (12.8%)	38 (15.7%)
50–64 years	38 (23.2%)	44 (18.2%)
65–74 years	34 (20.7%)	38 (15.7%)
75–84 years	31 (18.9%)	35 (14.5%)
> 85 years	14 (8.54%)	28 (11.6%)
Sex		
Man	112 (68.3%)	144 (59.5%)
Woman	52 (31.7%)	98 (40.5%)
Vaccination		
No	73 (44.5%)	117 (48.3%)
Yes	91 (55.5%)	125 (51.7%)
Vaccination status		
Not complete	83 (50.6%)	135 (55.8%)
Complete	81 (49.4%)	107 (44.2%)
Vaccination type		
No	73 (44.5%)	117 (48.3%)
Adenovirus-based	28 (17.1%)	29 (12.0%)
mRNA	63 (38.4%)	96 (39.7%)
First vaccination		
Pfizzer	54 (59.3%)	85 (68.0%)
Moderna	9 (9.89%)	11 (8.80%)
Janssen	18 (19.8%)	18 (14.4%)
Astrazeneca	10 (11.0%)	11 (8.80%)
Days from exposure* to survey date (median [IQR])	–	15 [4–51]

*For cases where only an interval of exposure is available, the middle point of this interval is taken as exposure date

**Fig. 2:**
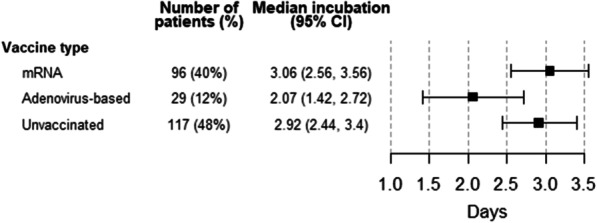
Median incubation time according to vaccine type

The unadjusted estimated median incubation period was 2.8 days (95%CI; from 2.5 to 3.1 days). The estimated 95^th^ percentile incubation period was 8 days, indicating that the probability that a patient has an incubation period of less than 8 days is 0.95. Additional file 1: Appendix 3 shows all the model combinations with their estimated coefficients, p-values and the AIC. We did not find any statistically significant differences in incubation periods stratifying by vaccination status, age, or sex, although we did find slight differences when stratifying by type of vaccine administered. The best model was the one that only includes the vaccine type as explanatory variable. The estimated median incubation period of the patients who received an adenovirus-based vaccine was 2.1 days (95%CI; from 1.4 to 2.7), which tended to be shorter than those of the patients not fully vaccinated or vaccinated with mRNA, which were 2.9 days (95%CI; from 2.4 to 3.4) and 3.1 days (95%CI; from 2.6 to 3.6), respectively. These differences did not reach statistical significance in the best model according to AIC, only adjusted by the type of vaccine. Table 2 and Fig. 2 show some percentiles and their 95% CIs according to type of vaccine and Table 3 contains the estimated model coefficients.

**Table 2:** Estimated percentiles of the incubation period

Percentile (days, 95%CI)	2.5th	25th	50th	75th	97.5th
Unadjusted estimates	0.5 (0.3, 0.7)	1.7 (1.5, 2.0)	2.8 (2.5, 3.1)	4.4 (3.9, 4.9)	9.8 (7.9, 11.7)
Vaccine types					
mRNA	0.6 (0.3, 0.8)	1.9 (1.6, 2.3)	3.1 (2.6, 3.6)	4.7 (3.9, 5.4)	10.3 (7.9, 12.7)
Adenovirus-based	0.4 (0.2, 0.6)	1.3 (0.9, 1.7)	2.1 (1.4, 2.7)	3.2 (2.2, 4.1)	7.0 (4.4, 9.4)
Unvaccinated	0.5 (0.3, 0.8)	1.8 (1.5, 2.2)	2.9 (2.4, 3.4)	4.4 (3.7, 5.2)	9.8 (7.7, 11.8)

**Table 3:** Best model estimates

	Estimate	Standard Error	p-value
Model parameters			
$log(\lambda )$	1.163	0.109	0.0000
$log(\phi )$	0.737	0.138	0.0000
$log(\rho )$	− 0.719	0.461	0.1186
Vaccine type			
mRNA	− 0.100	0.236	0.6714
Adenovirus-based	0.717	0.375	0.0558
Unvaccinated		(Reference group)	

The sensitivity analysis including only the patients with known date of exposure (censoring pattern I, n = 64) provided a similar unadjusted median to that obtained from the main analysis: 3.1 days, 95% CI from 2.5 to 3.8. The adjusted model with those 64 patients also estimates similar incubation median time for vaccinated patients [2.2 days (95%CI, 1.9–2.3) for adenovirus-based; and 3.1 days (95%CI, 2.7–3.6) for mRNA-vaccinated patients]; and for non-vaccinated patients [4.4 days (95%CI, 3.3–5.5)].

## Discussion

In south metropolitan area of Barcelona (Catalonia), the fifth wave of COVID-19 was associated with sustained community transmission: 52,756 cases registered in this area between the July 4th and December 21st of 2021.

The overall median incubation time with Delta variant is almost 3 days with a 95% percentile of about 8 days. The incubation times found in this study for COVID-19 are shorter than the observed with the earlier variants. For example, Quesada et al. [10] conducted a meta-analysis that concludes that the incubation time ranged from 5.6 to 6.7 days depending on the chosen model. There are few consolidated studies about the estimation of the incubation period for the more recent variants. One of them was the study of Du et al. [14], which reported an estimated median incubation period of 3.4 (slightly higher than our estimate) and 3.1 days for Delta and Omicron variants, respectively. Furthermore, a systematic review performed by Wu et al. [15] estimated a median of 4.4 and 3.4 days for these same variants.

Contrary to what was found in previous studies on severe acute respiratory syndrome [4, 5, 16], we have not found any evidence of a relevance of age on the incubation time.

This analysis suggests a shorter incubation period during the delta driven fifth wave with differences stratifying by type of vaccination administered, showing a shorter incubation period when administered adenovirus-based vaccines (i.e., AstraZeneca or Janssen) versus not fully vaccinated or mRNA vaccines. Despite further research is needed, this finding could be related to the different immune response produced by this type of vaccine [17, 18].

Our study has some limitations that should be acknowledged. First, the study cohort was made up of patients exclusively hospitalized for COVID-19 and did not include outpatient cases. This could cause a certain selection bias since hospitalized patients who had more severe symptoms could have a higher viral load and, therefore, shorter incubation times. Second, we did not dispose of some patient information that might be relevant for the incubation time, such severity of symptoms. We plan to address this issue in the future by fusing with other databases. Third, viral sequencing for confirmation of the variant in each case and viral load are not available. Following the European Commission indications [19] and the Spanish Health Ministry recommendations [20], a random sample between 5 and 10% (depending on the cumulative incidence) of the viral genome from the positive results were sequenced in our reference laboratory to obtain the variant. Since the data on the sequencing are referred anonymized to the national center we did not know the specific variant of each patient included in this study. However, during the study period we can confirm that more than 90% of the SARS-Cov-2 samples sequenced in this random sample were Delta variant, while the other 10% mainly corresponded to Alpha and Beta variants [21]. Fourth, the median of the elapsed time between the exposure and the completion of the survey was 15 days (Table 1). This large time interval could lead, on the one hand, to a low precision in the range of exposure dates and, on the other, to a certain recall bias, that is, patients with longer incubation times were more likely to have forgotten the date of their exposure. Fifth, the maximum incubation period of 14 days was based on previous literature. Although, there is no full consensus on this data, sensitivity analyses done with assumed longer maximum incubation periods (e.g., 21 days) did not lead to substantially different results of the ones described in our work.

Finally, the fact that a large percentage of patients (40%) were not even able to provide a partial interval of exposure could impact public health measures. This could either be because they did not remember having any contact, or were not aware of having been in any risk situation or, even because the patient had been continuously exposed to risk of infection. Although the planned sample size contemplated a 20% (instead of 40%) of non-responders, additional precision was gained due to the fact that there was a higher percentage of patients (16%) who remembered the exact date of exposure compared to previous studies; for example, in the article by Pak et al. [4], this percentage did not reach 5%. Table 1 shows that the differences between included and excluded patients regarding sex, age and vaccination status are modest and it is not anticipated that they may lead to any bias in the estimation of the incubation time.

## Conclusion

A thorough understanding of the distribution of the COVID-19 incubation time is essential for the control of this disease. One of the reasons why the knowledge of the distribution of the incubation time is important is its relation with the generation time, i.e., the time between infection events in an infector-infectee pair. We remark here that the estimation of the basic reproductive number, R_0_, of COVID-19 is based on the generation time [22]. Some web applications such as COVID19-world [23] use the incubation time to estimate the R_0_ of COVID-19 through this generation time. Knowing the distribution of the incubation time is key for establishing public health interventions such as timely isolation or contact tracing and quarantining. In addition, incubation time is a key input of several epidemiological models (e.g., SIR model). The percentiles provided in Table 2 as well as the model estimates from Table 3 can be useful in future apps or studies that require it.

## Supplementary Information

**Additional file 1.** Supplementary material that contains: 1) sample size calculations; 2) the case report form; and 3) estimates from all fitted models.

## Data Availability

The datasets generated and/or analysed during the current study are not publicly available to preserve the individual privacy of the participants but they are available from the corresponding author on reasonable request and with the agreement of the DIVINE group.
