# Supplementary material for: SARS-Cov-2 incubation period according to vaccination status during the fifth COVID-19 wave in a tertiary-care center in Spain: a cohort study
Source: BMC Infect Dis. 2022 Nov 9;22:828. doi: 10.1186/s12879-022-07822-4 (PMC9645305; doi:10.1186/s12879-022-07822-4)

Supplementary material

# Appendix 1. Sample size

Let $T$ be the incubation period (in days) defined as the time from the COVID-19 exposure to the development of the first symptom. Denote by $F\left( t \right)$ the cumulative distribution function of $T$ and by $I_{p}$ the *p^th^* percentile, that is, $p=F(I_{p})$.

We need to plan how many patients will be interviewed so that we will be able to estimate $F\left( t \right)$ and in particular $I_{p}$ with a precision of $d$ days. The required sample size $N_{p}$will depend on the percentile that we choose and the width of the interval.

For the purpose of this investigation and from a realistic point of view we aim to recruit enough patients to estimate the $75\% (p=0.75)$ percentile with a precision of 2 days. As a consequence, the median of the incubation time will also be estimated with a precision $<2$ days.

Assume that based on our observed data we would be able to estimate the percentiles $\hat{I}_{p}$ and that, asymptotically would behave as a normal, that is, $\hat{I}_{p}\sim N\left( I_{p},\sigma_{p}^{2} \right).$ From these we could provide confidence intervals: $\left[ \hat{I}_{p}-z_{\alpha/2}\cdot\sigma/\sqrt{n} , \hat{I}_{p}+z_{\alpha/2}\cdot\sigma/\sqrt{n} \right]$with length $2z_{\alpha/2}\cdot\sigma/\sqrt{n}$ (Machin et al., 2011).

Based on our own computations from Wuhan data, and after fitting our data to other incubation studies, we can provide an estimate for $\sigma_{p}^{2}$, for the median $(p=0.5)$, and for the $75\% (p=0.75)$ percentile, and based on that $d=z_{\alpha/2}\cdot\hat{\sigma}_{p}/\sqrt{N_{p}}$, from were $N_{p}=z_{\alpha/2}^{2}\cdot\hat{\sigma}_{p}/d^{2}$. The estimated values for $\sigma_{p}^{2}$are quite large since they have been based on complex unprecise data and include the inherent variability due to the random censoring intervals.

**Table S1**: Estimated quantiles of the incubation period in two age groups (patients aged ≤ 42 years and patients aged > 42 years).

| **Percentile** | ${\hat{\boldsymbol{I}}}_{\boldsymbol{p(\leq42)}}$ | ${\hat{\boldsymbol{I}}}_{\boldsymbol{p(>42)}}$ | ${\hat{\boldsymbol{\sigma}}}_{\boldsymbol{p(\leq42)}}^{\boldsymbol{2}}$ | ${\hat{\boldsymbol{\sigma}}}_{\boldsymbol{p(>42)}}^{\boldsymbol{2}}$ | ${\hat{\boldsymbol{\sigma}}}_{\boldsymbol{p(pooled)}}^{\boldsymbol{2}}$ |
| --- | --- | --- | --- | --- | --- |
| 2.5^th^ | 0.6 (0.3, 0.9) | 0.8 (0.4, 1.2) | - | - | - |
| 25^th^ | 0.9 (2.3, 3.5) | 3.9 (3.0, 4.7) | - | - | - |
| 50^th^ | 5.3 (4.3, 6.3) | 7.0 (5.6, 8.3) | 40.6 | 74.0 | 57.3 |
| 75^th^ | 8.7 (7.0, 10.4) | 11.6 (9.4, 13.8) | 117.4 | 196.5 | 157.0 |
| 97.5^th^ | 20.3 (15.2, 25.5) | 27.0 (20.3, 33.7) | 1077.0 | 1822.9 | 1450.0 |

Since our aim is to provide precise 95% confidence intervals for several population groups (e.g., two age groups), the sample sizes provided in Table 2 should be increased according to those and the underlying model. Our pragmatic goal here is to estimate the $I_{0.75}$ for a precision of 2 days (interval width=4). Assuming 20% of non-responders, we need to recruit 378 patients to estimate $I_{0.75}\pm2$ days in two different groups. With a constant accrual rate equal to 2 patients/day and 40% of patients belonging to the smallest group, the recruitment period will extend from 1st July 2021 to 22nd February, 2022, that is 236 days to achieve the desired sample size (189) in the smallest of the two groups.

**Table S2**: Estimated sample size without (left) and with (right) considering a 20% of non-responders according to a specific precision (columns).

| **Percentile** | **± 1 day** | **± 2 days** | **± 3 days** |
| --- | --- | --- | --- |
| 50th | 221/276 | 56/69 | 34/42 |
| 75th | 604/754 | 152/189 | 68/84 |
| 97.5th | 5,571/6,963 | 1,393/1,741 | 620/774 |

#
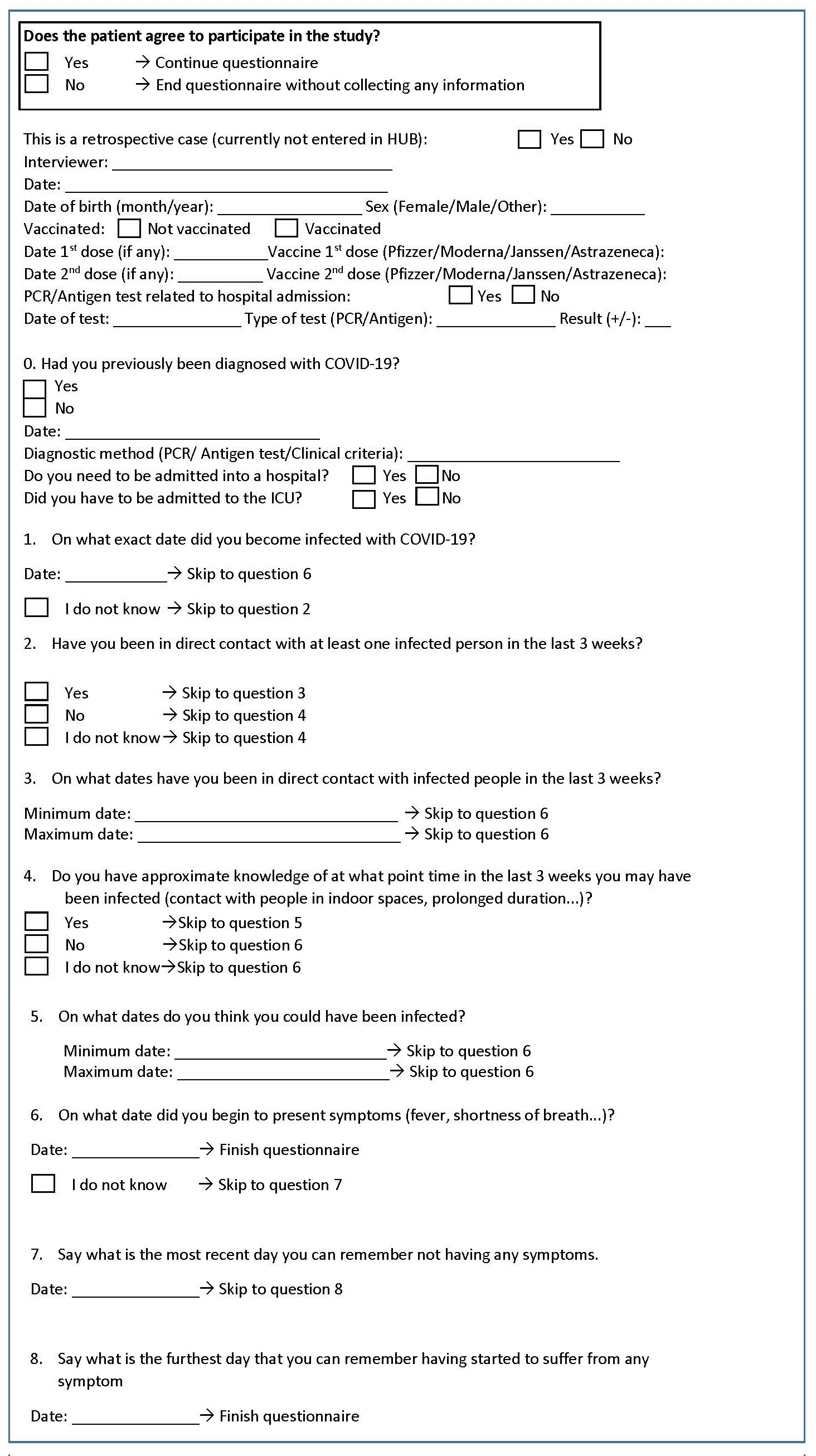
Appendix 2. Case Report form

# Appendix 3. All fitted models

This appendix shows some output of all the models tested according to sex, age and vaccination. Three variables related to vaccination were separately considered to avoid collinearity:

- *vaccine*: if the patient had been vaccinated with at least one dose (No/Yes)
- *vaxstat*: vaccination status (Not complete [0-1 dose]/ Complete [2 doses])
- *vaxtype*: vaccination type (No/AdenoVirus/mRNA)

**Point estimates**

Table S3 shows the point estimates associated to each parameter in each model (first column).

**Table S3**: Estimated coefficients in all the models tested. The last column shows the Akaike information criteria (AIC) for each model. In bold, selected model. λ, $\phi,$ρ are the distribution parameters.

| Model | $log\left( \hat{\lambda} \right)$ | $log\left( \hat{\phi} \right)$ | $log\left( \hat{\rho} \right)$ | $\beta_{Sex=W}$ | $\beta_{Age}$ | $\beta_{Vax=Yes}$ | $\beta_{Vax=Complete}$ | $\beta_{Vax=Adeno}$ | $\beta_{Vax=mRNA}$ | AIC |
| --- | --- | --- | --- | --- | --- | --- | --- | --- | --- | --- |
| ~1 | 1.137 | 0.693 | -0.759 |  |  |  |  |  |  | 547.839 |
| ~sex | 1.188 | 0.661 | -0.837 | 0.221 |  |  |  |  |  | 548.651 |
| ~age | 1.112 | 0.724 | -0.637 |  | 0.025 |  |  |  |  | 549.860 |
| ~vaccine | 1.173 | 0.701 | -0.733 |  |  | 0.134 |  |  |  | 549.566 |
| ~vaxstat | 1.134 | 0.722 | -0.652 |  |  |  | 0.069 |  |  | 549.806 |
| **~vaxtype** | **1.163** | **0.737** | **-0.719** |  |  |  |  | **0.717** | **-0.100** | **544.563** |
| ~sex + age | 1.156 | 0.725 | -0.656 | 0.212 | 0.012 |  |  |  |  | 550.794 |
| ~sex + vaccine | 1.260 | 0.635 | -1.015 | 0.209 |  | 0.207 |  |  |  | 550.692 |
| ~sex + vaxstat | 1.228 | 0.684 | -0.807 | 0.238 |  |  | 0.058 |  |  | 550.693 |
| ~sex + vaxtype | 1.231 | 0.682 | -0.822 | 0.281 |  |  |  | 0.764 | -0.023 | 545.045 |
| ~age + vaccine | 1.211 | 0.663 | -0.870 |  | -0.004 | 0.161 |  |  |  | 551.747 |
| ~age + vaxstat | 1.133 | 0.696 | -0.807 |  | 0.040 |  | -0.008 |  |  | 552.039 |
| ~age + vaxtype | 1.152 | 0.710 | -0.674 |  | 0.067 |  |  | 0.768 | -0.199 | 546.583 |
| ~sex + age + vaccine | 1.274 | 0.687 | -0.853 | 0.325 | -0.028 | 0.165 |  |  |  | 552.272 |
| ~sex + age + vaxstat | 1.258 | 0.656 | -0.952 | 0.198 | -0.052 |  | 0.224 |  |  | 552.888 |
| ~sex + age + vaxtype | 1.229 | 0.727 | -0.637 | 0.275 | -0.031 |  |  | 1.023 | -0.050 | 547.730 |
|  |  |  |  |  |  |  |  |  |  |  |

**P-values**

Table S4 shows the p-values associated to the contrast with null hypothesis that the corresponding parameter is zero.

**Table S4**: p-values of each coefficient for all the models tested. In bold, selected model. λ, $\phi,$ρ are the distribution parameters.

| Model | $log\left( \hat{\lambda} \right)$ | $log\left( \hat{\phi} \right)$ | $log\left( \hat{\rho} \right)$ | $\beta_{Sex=W}$ | $\beta_{Age}$ | $\beta_{Vax=Yes}$ | $\beta_{Vax=Complete}$ | $\beta_{Vax=Adeno}$ | $\beta_{Vax=mRNA}$ |
| --- | --- | --- | --- | --- | --- | --- | --- | --- | --- |
| ~1 | 0 | 0 | 0.090 |  |  |  |  |  |  |
| ~sex | 0 | 0 | 0.078 | 0.304 |  |  |  |  |  |
| ~age | 0 | 0 | 0.126 |  | 0.834 |  |  |  |  |
| ~vaccine | 0 | 0 | 0.084 |  |  | 0.533 |  |  |  |
| ~vaxstat | 0 | 0 | 0.115 |  |  |  | 0.763 |  |  |
| **~vaxtype** | **0** | **0** | **0.119** |  |  |  |  | **0.056** | **0.671** |
| ~sex + age | 0 | 0 | 0.140 | 0.360 | 0.917 |  |  |  |  |
| ~sex + vaccine | 0 | 0 | 0.109 | 0.311 |  | 0.315 |  |  |  |
| ~sex + vaxstat | 0 | 0 | 0.066 | 0.272 |  |  | 0.790 |  |  |
| ~sex + vaxtype | 0 | 0 | 0.097 | 0.196 |  |  |  | 0.047 | 0.942 |
| ~age + vaccine | 0 | 0 | 0.052 |  |  | 0.427 |  |  |  |
| ~age + vaxstat | 0 | 0 | 0.128 |  | 0.715 |  | 0.939 |  |  |
| ~age + vaxtype | 0 | 0 | 0.081 |  | 0.623 |  |  | 0.043 | 0.494 |
| ~sex + age + vaccine | 0 | 0 | 0.095 | 0.139 | 0.815 | 0.505 |  |  |  |
| ~sex + age + vaxstat | 0 | 0 | 0.100 | 0.372 | 0.723 |  | 0.428 |  |  |
| ~sex + age + vaxtype | 0 | 0 | 0.121 | 0.244 | 0.826 |  |  | 0.011 | 0.865 |

**Figures S1:** Forest plot of the full model with age, sex and vaccination type representing the 95% CI for the median incubation time.


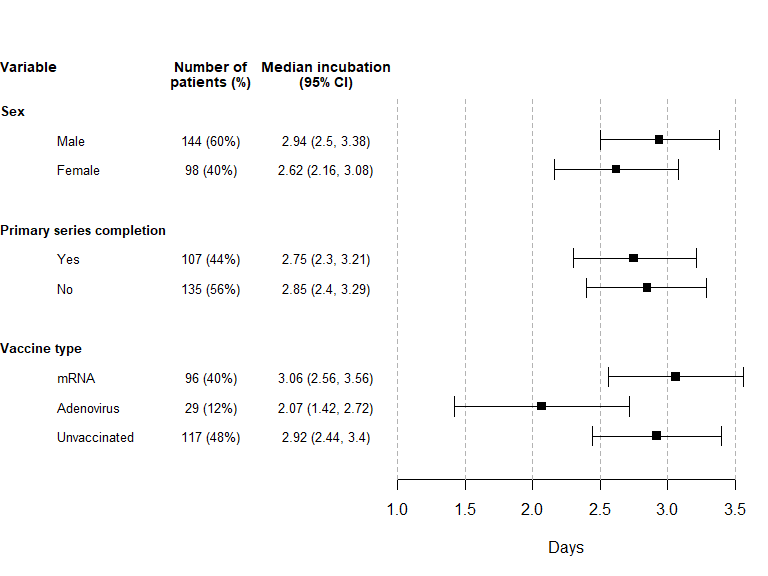

Supplement: Supplementary file 1 — Additional file 1. Supplementary material that contains: 1) sample size calculations; 2) the case report form; and 3) estimates from all fitted models. [file 12879_2022_7822_MOESM1_ESM.docx]
